# Book Review

**Published:** 1893-03

**Authors:** 


*Notes on Pathology*. By the late R. E. CARRINGTON, M.D. Lond., F.R.C.P. Pp. 150. London: H. K. LEWIS. 1892.—This little book is a reprint of notes which were distributed by the late Dr. Carrington to students attending his demonstrations on pathological anatomy. They have been collected from the pages of *Guy's Hospital Gazette*, edited by Dr. Crook and Mr. Mackeson, and issued in this volume with a delightful little preface by Dr. Goodhart. The organs are taken one by one, and the various lesions shortly described in a systematic manner, so that they may easily be remembered. These descriptions, though brief, are admirably clear. For instance, we would call attention to the account given of the lesions found in the various forms of Bright's disease. Though in many respects these *Notes* may be looked upon as a condensed *Wilks and Moxon*—that best of all books for general *post-mortem* and museum work,—yet, insomuch as that work is much too large for ordinary students, no better book than this can be put in the hands of pathological clerks.

*The Transactions of the Pathological Society of Manchester for the Session* 1891-92. Edited by J. N. KELYNACK, M.B. Vol. I.—This volume contains 162 pages, recording work done at eight meetings under the presidency of Dr. T. C. Railton. No less than seventy-six independent topics are dealt with: clearly the Society wastes no time in superfluous or irrelevant talk, and the transactions are a good record of a good winter's work.

*Du Traitement par l'Électrolyse des Déviations et Éperons de la Cloison du Nez*. Par J. BERGONIÉ et E. J. MOURE. Pp. 67. Paris: O. DOIN. 1892.—This pamphlet is well worth reading by those who are called upon to deal with abnormalities of the nasal septum. Electrolysis in the authors' hands has been of much value in such cases, and is free from the drawbacks incidental to some other methods.

*Differentiation in Rheumatic Disease (so-called)*. By HUGH LANE, L.R.C.P., M.R.C.S. Second Edition. Pp. ix., 121. London: J. & A. CHURCHILL. 1892.—A second edition of this book has been quickly demanded. The author has attempted to give, what has long been needed, a series of clearly defined differentiations between the various affections commonly and loosely described as rheumatic gout. He believes that chronic rheumatoid arthritis has no connection whatever with acute rheumatism, but that its constitutional cause is a combination of the hereditary taints of gout and phthisis. Rheumatic arthritis is, on the other hand, caused by debility, the debilitating cause being unquestionably rheumatism; the disease is confined to the joints, nervous symptoms are absent, the heart is often diseased, and adults mostly over middle age are its victims. The chapters on treatment are well worthy of careful reading: they show that we are not so helpless as is commonly believed, and that the mineral waters of Bath are deserving of their long-established reputation.

*Health. The Voyage to South Africa, and Sojourn There.* Pp. 80. London. 1891.—This volume consists of a series of essays collected and republished by the Castle Mail Packets Company for the International Congress of Hygiene and Demography of 1891. It comprises extracts from the Official Handbook of the Cape of Good Hope, edited by John Noble, and a lecture on South Africa as a health-resort by Dr. E. Symes Thompson. It contains much valuable information on the country, its climate, and its suitability as a resort for invalids.

*Dermic Memoranda.* By WILLIAM GEMMELL, M.B. Pp. 116. London: BAILLIERE, TINDALL, and COX. 1892.—We have looked carefully through this book, and, upon the whole, consider that the arrangement of the matter is good, the separate page embodying treatment is concise and readily consulted, and that the endeavour of the author to demonstrate to the *student* is very fairly carried out. The definition of terms on primary lesions of the skin is clear and scientific. In a book of this size there must, of course, be many omissions; but we are rather surprised that not a word is said about salicylic acid in the treatment of eczema, nor of chrysophanic acid in that of psoriasis; nor is mention made of ichthyol and its derivatives.

*The Australasian Medical Directory and Handbook.* Edited by LUDWIG BRUCK. Third Edition. Sydney: The *Australasian Medical Gazette* Office. 1892.—The first edition of this work was published in 1883. The present volume contains the names of all known legally-qualified physicians, surgeons, and general practitioners resident in Australia, Tasmania, New Zealand, Fiji, Samoa, Tonga, and New Guinea, with their addresses, qualifications, past and present appointments, together with an obituary of doctors dead since the publication of the second edition in July, 1886. The book also gives an alphabetical list of post towns, with the geographical peculiarities of each, and the names of practitioners residing in them. The Medical Acts of Australasia are well summarised, and a list of the constitution and work of the numerous medical and allied societies is added. The scale of medical and surgical fees is worthy of study, as these are distinctly in advance of those usually charged in this country.

*The Clinical Journal.* London: E. KNIGHT.—This *Journal* has commenced its career under most favourable auspices. All its articles up to the present are written by authorities on the subjects upon which they dilate, and the subjects chosen have been of broad and general interest. The busy practitioner may expect in each issue of this new weekly to find practical help, as well as scientific information.

*Index-Catalogue of the Library of the Surgeon-General's Office, United States Army.* Vol. XIII., Sialagogues—Sutugin. Washington: Government Printing Office. 1892.—We cordially welcome another instalment of this magnificent undertaking, the full value of which is appreciated by those workers in matters medical who are familiar with it. We have recently, with a considerable amount of dismay, noticed statements appearing in our American contemporaries to the effect that the official grants in aid of this work are to be curtailed or withdrawn. Anything which would cripple the resources of those engaged in it would be a world-wide calamity. As we write, the re-assuring statement is made that the national subsidy is to be continued.

*The Year-Book of Treatment for* 1893. Pp. 496. London: Cassell and Company, Limited.—The comparatively early appearance of this well-known annual will be welcomed by all practitioners, and more especially by those who, being desirous of keeping themselves *au courant* with new methods of treatment in every department of medicine and surgery, are compelled by the exigencies of busy practice to avail themselves of such works as the one before us. In the present edition the *Year-Book* reaches its ninth year, and in its general arrangement it differs but little from issues of previous years. Two new articles are added, however: one on "Anæsthetics" by Dr. Dudley Buxton; and there is also a separate article by Prof. W. H. Corfield, M.D., on "Public Health and Hygiene." The other sections are entrusted to the same authors as in the last edition; amongst whom we are glad to find again the names of Dr. E. Markham Skerritt, who writes on diseases of the lungs and respiration, and Dr. Barclay Baron, who is responsible for the summary of new treatment in diseases of the throat and nose. The introduction of woodcuts, chiefly in the surgical sections, adds to the interest and clearness of the text in many places.

*The Medical Annual.* Pp. 767. Bristol: John Wright & Co. 1893.—This volume is a worthy successor of those that have preceded it. The amount of information that is compressed into it is really astonishing, and it must be of great service to the busy practitioner. The way in which the book is turned out of hand, as regards paper, type, and illustrations, reflects great credit on the local firm which publishes it. As in the *Year-Book*, doctors connected with Bristol are well represented. Dr. Shingleton Smith treats of pulmonary diseases, and Dr. Watson Williams of diseases of the throat. The subject of general medicine is entrusted to Dr. F. J. Wethered, an old Bristol student. We recommend the volume to those who, from lack of time or opportunity, are unable to read the numerous journals which are laid under contribution in collecting the fund of information contained in it.

*The Journal of Pathology and Bacteriology.* Vol. I., No. 3. February, 1893. Edinburgh: YOUNG J. PENTLAND.—In this, the third number of the new journal, are so many good articles that it seems invidious to pick out any for special mention; we may, however, perhaps refer to one or two of more particular interest. Dr. Sherrington, the well-known physiologist, recently appointed Brown Professor, shows in an elaborate research that Cohnheim's view—that the body protects itself, in the event of bacterial invasion, by secreting living germs through the kidneys and liver—is probably untrue; for he finds that non-pathogenic bacteria injected into the blood do not appear in the urine and bile, whilst if injected pathogenic bacteria are found in these secretions, there is evidence, in the presence of either blood or albumen, that the secretory epithelium is no longer normal; so that he concludes that the healthy secreting epithelium is impervious to micro-organisms, and only becomes so after soluble poisons produced by them have had time to act on it. Dr. Lockhart Gillespie, of Edinburgh, who had previously shown that by far the larger part of the hydrochloric acid secreted into the stomach rapidly forms proteid-hydrochlorides by entering into combination with proteids in the food, here demonstrates that these proteid-hydrochlorides have a much less powerful bactericidal action than has free hydrochloric acid. The presence, then, of bacteria in the intestines seems much less inexplicable than formerly. Finally, we may mention that there is an elaborate account of the pathology of the pituitary body by Messrs. Boyce and Beadles.

*The Advertiser's A B C.* Pp. clxxiv., 17—959. London: T. B. BROWNE. 1893.—This book, although mainly intended as a guide to advertisers, will be found of great interest by all readers of newspapers or magazines. It contains lists of these arranged under the towns of publication, and in most cases accompanied with interesting information concerning them. Not the least useful part of the book is the classified index of class and trade journals. We are, however, at a loss to understand why our own *Journal*, which duly appears in other "indices" (why not "indexes"?) should not be given under "magazines and periodicals," p. lxxv. In the "Medical" list there are some noteworthy omissions. It is clear that this part of the work has not passed under a medical eye; for the *British Gynæcological Journal* appears by itself under "Gynæcology," but has no place under "Medical;" while the *Ophthalmic Review* has not only a place under that heading, but is also given by itself under "Ophthalmy" (*sic*). It is a positive pleasure to look through this book, not only on account of the information which it conveys, but also because of the admirable way in which it is printed and arranged.

